# SECONDARY ROLE STRAIN AND GRIEF OF DEMENTIA FAMILY CAREGIVING AS PREDICTORS OF BURDEN

**DOI:** 10.1093/geroni/igad104.2556

**Published:** 2023-12-21

**Authors:** Hannah Mason, Francesca Falzarano

**Affiliations:** Fordham University, New York City, New York, United States; Weill Cornell Medicine, New York City, New York, United States

## Abstract

Services often aim to improve caregiver experiences for dementia family caregivers by addressing challenges in daily care activities and enhancing care quality for care-recipients. Supporting caregivers should not exclusively focus on supporting activities related to direct care provision (e.g., primary stressors), but should also include services that holistically support the caregiver’s well-being. Identifying an individual’s unique needs beyond their caregiving role is crucial; the growing phenomenon of caregiver burnout, burden, and psychosocial struggle includes experiencing a loss of identity, increased desire to escape, and intense role captivity. While some services are more individualized, such as one-on-one therapy, there is an overall gap in caregiver support in secondary domains. The current study examined whether primary stressors (e.g., activities of daily living and everyday care tasks) or secondary stressors (e.g., strain on other relationships and responsibilities) were more predictive of burden in a sample of 46 participants who completed a mixed-method study examining the heterogenous experiences and unmet needs of dementia family caregivers. Analysis shows that interference of the care role with responsibilities to other family (i.e., secondary role strain) and feeling pre-loss grief over their loved one’s illness were significant predictors of greater burden compared to primary stressors. Results indicate the importance of addressing secondary stressors such as pre-loss grief and deterioration of one’s microenvironment (including social support, familial relationships, and identity) in intervention efforts to better enhance quality of life and well-being in dementia care.

